# Association between adjusted body size index and abdominal aortic calcification among US older adults aged 40 years and above from a cross-sectional survey of the NHANES 2013-2014

**DOI:** 10.3389/fendo.2024.1411701

**Published:** 2024-09-23

**Authors:** Yutong Chen, Yi Ding, Shanliang Jin, Yanwei Zhang

**Affiliations:** Department of Anesthesiology, Shanghai Ninth People’s Hospital, Shanghai Jiao Tong University School of Medicine, Shanghai, China

**Keywords:** adjusted body size index, abdominal aortic calcification, cross-sectional survey, elderly, NHANES

## Abstract

**Purpose:**

This research aimed to assess the correlation between the Adjusted Body Shape Index (ABSI) and the presence of abdominal aortic calcification (AAC) among middle-aged and older American adults.

**Methods:**

Employing a cross-sectional design, this study analyzed data from the 2013-2014 National Health and Nutrition Examination Survey (NHANES), focusing on 3077 participants aged 40 and above. AAC detection was conducted using dual-energy X-ray absorptiometry (DXA). ABSI was determined based on waist circumference (WC), weight, and height data. The association between ABSI and AAC was examined through multiple linear regression, smoothed curve analysis, threshold effect evaluation, subgroup analysis, and interaction testing.

**Results:**

The study encompassed 3077 individuals aged 40 and above. Findings indicated a noteworthy positive relationship between ABSI and AAC when adjusting various covariates. Analysis of threshold effects identified a K-point at 0.0908, showing no significant effect to its left but a significant effect to its right. Further, subgroup and interaction analyses highlighted the ABSI-AAC connection specifically within different age groups and among individuals with diabetes.

**Conclusion:**

Higher ABSI was correlated with higher AAC score.

## 1 Introduction

Vascular calcification is characterized by the accumulation of calcium salts in the blood vessel walls, leading to stiffness and loss of elasticity. It is established that the calcification of coronary arteries is a predictor for the risk of cardiovascular diseases and mortality (1). Despite the commonality of calcification in vascular areas beyond the coronary arteries, the prognostic value of such calcifications has been less explored. Notably, the abdominal aorta is among the first vessels to experience calcification, typically before the coronary arteries (2, 3). The incidence of AAC is around one-third in individuals between 45 to 54 years of age and escalates to as much as 90% in those 75 years and older. For elderly patients with type 2 diabetes(T2DM) or chronic kidney disease (CKD) requiring dialysis, prevalence figures range from 84% to 97%. The link between AAC and cardiovascular events is well-documented. In individuals aged 45 to 84 years, AAC has been shown to independently correlate with cardiovascular disease mortality and is more strongly related to total mortality compared to coronary artery calcification (4). Severe AAC in older white women has been closely linked to an increased risk of atherosclerotic disease events and decreased long-term survival (5). Furthermore, AAC has been identified as an independent predictor for myocardial infarction and cardiovascular events (6). Particularly in populations with a high prevalence of AAC, such as older adults and individuals with chronic kidney disease, the presence of AAC significantly heightens the risk of future cardiovascular events and suggests a poorer prognosis. Highlighting the importance of AAC is crucial for clinicians to effectively assess and manage the cardiovascular risk of their patients (7).

Obesity has escalated into a widespread chronic condition worldwide. Data from 2015 indicate that around 603.7 million adults worldwide are classified as obese. Since 1980, obesity rates have doubled in over 70 countries and continue to rise elsewhere. A high BMI is linked to over 4 million deaths annually on a global scale, with cardiovascular diseases accounting for the majority of these fatalities (8). The global healthcare cost of obesity is estimated at $2 trillion (9). Notably, the location of body fat accumulation is variably linked to obesity-related health outcomes, with metabolic complications of obesity showing a direct association with the extent of abdominal fat (10). Although the World Health Organization (WHO) uses BMI to assess general obesity, there is no consensus on how to assess the distribution of body fat. ABSI is independent of BMI (11), and complements the optimal BMI by effectively risk stratifying between underweight, obese, and normal-weight and overweight BMI categories (12). The ABSI estimates both visceral abdominal fat and general overall adiposity and is a better predictor of premature mortality (13).

While the link between Body Mass Index (BMI) and the risk of cardiovascular disease has been the subject of considerable research, studies focusing on the ABSI are notably less common. The relationship between ABSI and AAC particularly lacks emphasis in existing research. This investigation seeks to delve into the possible correlation between ABSI and AAC, drawing upon data from NHANES. It aims to enrich the framework for evaluating cardiovascular disease risk by incorporating insights into the intricate interplay between body fat distribution and cardiovascular health. Moreover, by uncovering the potential connection between ABSI and AAC, this study contributes to a deeper comprehension of obesity’s impact on the cardiovascular system. Such insights are crucial for informing the development of future clinical practices and public health initiatives.

## 2 Methods

### 2.1 Study population

**Figure 1:**
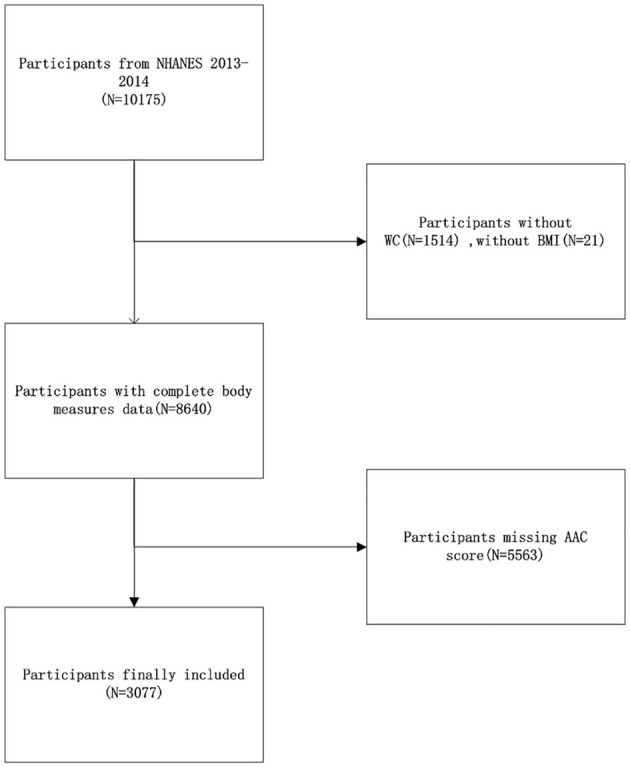
Flow chart showing the NHANES participants’ selection.

NHANES is a cross-sectional analysis aimed at gathering essential health status data and information, employing a stratified, multi-stage probability sampling method within the non-institutionalized demographic. The National Center for Health Statistics (NCHS) is responsible for executing the survey. NCHS’s ethical review committee has approved the study protocol, and all involved participants provided written informed consent. For this research, the 2013 to 2014 NHANES dataset was utilized, selected specifically because it was the only survey period to AAC measurements. Initially, 10,175 individuals were screened in this survey. The study excluded 1514 participants due to the absence of waist circumference data, 21 participants for lacking BMI data, and 5563 participants for missing AAC data. Consequently, 3077 participants were ultimately included in this analysis (**Figure 1**).

### 2.2 ABSI

ABSI is designed to be a dimensionless index. Although the absolute value of the ABSI is not a direct measure of obesity, it can be used in statistical analyses to compare the relative risk of abdominal obesity between different individuals or groups. ABSI was calculated by the following equal:


$$ABSI\;=\;WC\;/\;\left(BMI^{2/3}\;*\;Height^{1/2}\right).$$

### 2.3 Measurement of AAC

AAC was assessed using dual-energy X-ray absorptiometry (DXA). During the 2013-2014 NHANES cycle, lateral DXA scans of the thoracolumbar spine were conducted on participants aged 40 and above at the Mobile Examination Center (MEC). Individuals were excluded from DXA scanning if they were pregnant, had used a radiographic contrast agent (e.g., barium) within the last seven days, reported a weight over 450 pounds, or had scoliosis treated with Harrington rods. The scans utilized a Hologic Discovery Model A densitometer, produced by Hologic, Inc., based in Marlborough, Massachusetts, operating on Apex 3.2 software. For evaluating AAC, the AAC24 rating system was employed, dividing the anterior and posterior aorta walls into four segments each, located in front of the lumbar vertebrae from L1 to L4. These eight segments allow for the visual identification of aortic calcification as white spots or lines across the aorta’s walls. Calcification scoring is detailed as follows: a score of 0 indicates no calcification; 1 signifies calcification occupying a third or less of the segment’s aortic wall; 2 denotes calcification extending over a third but under two-thirds of the wall; and 3 reflects more than two-thirds coverage. Each anterior and posterior aortic wall is scored independently, leading to a potential range of 0 to 6 per lumbar vertebra and a cumulative possible score ranging from 0 to 24. Additionally, the AAC8 scale was applied to quantify the total calcification length along the anterior and posterior walls of the aorta across vertebrae L1 to L4. The scoring criteria are as follows: 0 for no observable calcification; 1 if total calcification length is up to one vertebral bone height; 2 for length surpassing one but not two vertebral heights; 3 for calcification extending beyond two but under three vertebral heights; and a score of 4 is assigned if the calcification length exceeds three vertebral bone heights.

### 2.4 Covariates

In this study, we included multiple characteristics as covariates for analysis, including age, gender, race, education level, income level (measured by the Poverty Income Ratio, PIR), smoking history, frequency of alcohol intake, diabetes, and the presence of hypertension. Diabetes and hypertension were based on participants’ self-reported responses to the questions, “Doctor told you have diabetes” and “Ever told you had high blood pressure” These data, along with the analytical methods, were obtained from the publicly available NHANES database, which is widely used for research purposes. All these covariates were included in the fully adjusted multivariable regression models.

Missing data were present in several covariates with an overall missing rate of 0.76%. We performed random forest imputation using the R package missForest to handle missing values, which has been shown to outperform traditional methods such as mean imputation and multiple imputation for small amounts of missing data. Sensitivity analysis using complete-case analysis yielded nearly identical results (data not shown). After imputation, the dataset was complete, and all analyses were based on the fully imputed data.

### 2.5 Statistical analysis

In this research, the application of NHANES sampling weights was meticulously adhered to for all statistical analyses, aligning with the CDC’s guidelines and acknowledging the intricacies of the survey’s multi-stage, complex sampling design. Statistical evaluations were conducted utilizing EmpowerStats software (version 4.2) and the R statistical package (version 4.2), ensuring rigorous analysis. Participants were divided into three equal tertiles by ABSI scores. Demographic differences were examined using chi-square tests for categorical variables and weighted t-tests for continuous variables. Since AAC scores were non-normally distributed with many zero values, we applied natural logarithmic transformation (ln(AAC8 + 1) and ln(AAC24 + 1)) to the outcome variables, which satisfied the normality assumption of linear regression. Multivariate linear regression was used to assess the association between ABSI and AAC. All regression coefficients reported in the results are based on the natural log-transformed AAC scores. Additionally, a weighted smoothing curve-fitting method alongside a threshold effect analysis was utilized to investigate the non-linear dynamics of the ABSI-AAC relationship. Subgroup analyses and interaction tests were further implemented to delve into the specifics of this association across different demographics. The threshold for deeming results statistically significant was established at a two-tailed p-value of less than 0.05.

## 3 Results

### 3.1 Baseline characteristics

**Table 1** outlines the demographic characteristics of the study participants, who were segmented into three groups based on tertiles of their ABSI. The study included 3077 adults aged 40 years and above. The mean age of the participants was 57.36 ± 11.50 years, with a gender distribution of 48.20% male and 51.80% female. The thresholds for the ABSI tertiles were identified as 0.08102 and 0.08489. Comparing the higher to the lower ABSI group reveals several notable differences: the high ABSI group had an older average age, a greater percentage of male participants, a higher likelihood of being non-Hispanic white, a lower Poverty Income Ratio (PIR), a lower educational attainment level, a higher proportion of current smokers, and a higher prevalence of individuals with diabetes and hypertension.

**Table 1:** Weighted characteristics of the study population based on ABSI tertiles.

Characteristics	Adjusted body size index			P value
	T1 (ABSI ≤ 0.08102)	T2 (0.081021 < ABSI ≤ 0.08489)	T3 (ABSI > 0.08489)	
Age (years)	52.72 ± 9.90	56.63 ± 11.10	63.11 ± 11.04	<0.0001
Gender (%)				<0.0001
Male	36.49	51.69	57.15	
Female	63.51	48.31	42.85	
PIR	3.34 ± 1.59	3.16 ± 1.59	2.99 ± 1.61	<0.0001
Race (%)				<0.0001
Mexican American	7.03	7.91	5.84	
Other Hispanic	4.56	5.91	3.61	
Non-Hispanic White	66.14	69	78.18	
Non-Hispanic Black	14.66	9.29	6.27	
Non-Hispanic Asian	5.16	5.61	4.57	
Other Race	2.46	2.27	1.53	
Education (%)				0.001
< high school	4.04	5.13	6.23	
9-11th grade	8.75	9.87	12.22	
High school graduate	20.34	22.64	22.1	
Some college or AA degree	27.85	30.55	30.61	
College graduate or above	39.02	31.81	28.84	
Don’t Know			0.05	
Smoked ≥ 100 cigarettes (%)				<0.0001
Yes	36.76	46.59	55.24	
No	63.24	53.41	44.76	
Alcohol intakes ≥12drinks/year (%)				0.6075
Yes	78.75	78.47	77.04	
No	63.24	53.41	44.76	
HBP (%)				<0.0001
Yes	39.35	37.57	54.83	
No	60.65	62.43	45.17	
Diabetes (%)				<0.0001
Yes	6.91	13.11	19.27	
No	90.77	83.07	75.71	
Broadline	2.31	3.82	5.03	
AAC24	0.78 ± 2.24	1.33 ± 3.00	2.32 ± 4.14	<0.0001
AAC8	0.38 ± 0.91	0.58 ± 1.12	0.94 ± 1.47	<0.0001

Mean ± SD for continuous variables: the P value was calculated by the weighted linear regression model; (%) for categorical variables: the P value was calculated by the weighted chi-square test.

T tertiles, PIR Ratio of family income to poverty, BMI Body mass index, HBP High blood pressure, AAC abdominal aortic calcification.

### 3.2 Relationship between ABSI and AAC

**Figure 2:**
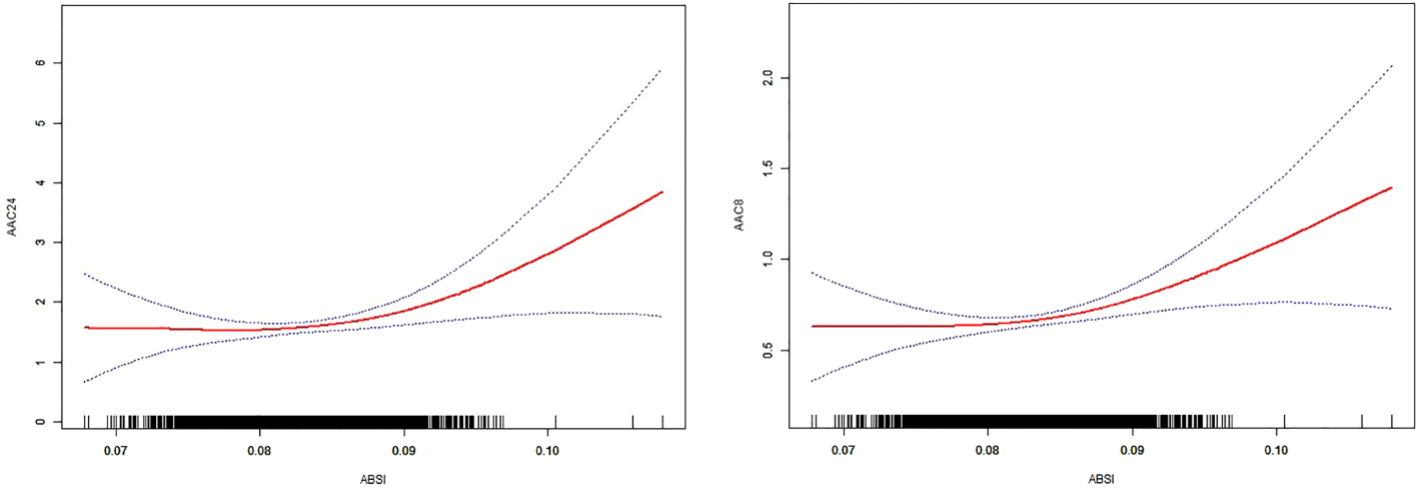
The solid red line represents the smooth curve fit between the variables. The 95% confidence interval derived from the fit is shown by blue bands.

**Table 2** presents the findings from the multifactor regression analysis, illustrating the relationship between the ABSI and AAC scores. In the unadjusted model, ABSI exhibited a strong positive correlation with AAC24, with a coefficient [β=154.34, (confidence interval: 129.66, 179.02)], indicating a significant association. This significant positive correlation persisted in Model 2, even after adjusting for gender, age, and race [β=48.01, (22.06, 73.96)]. In Model 3, which additionally included education level, poverty income ratio (PIR), hypertension, diabetes, smoking status, and alcohol intake, each 0.01 unit increase in ABSI was associated with a 0.0505 increase in ln(AAC8 + 1) (β=5.05 per 0.01 unit, 95% CI: 0.77–9.33, P = 0.0208) and a 0.0670 increase in ln(AAC24 + 1) (β=6.70 per 0.01 unit, 95% CI: 0.32–13.08, P = 0.0397). When ABSI was divided into tertiles, no significant linear trend was observed in the fully adjusted model. Despite dividing ABSI into tertiles and testing for trends with AAC, no significant trend emerged. However, further exploration through smoothed curve fitting and threshold effect analysis revealed a nonlinear relationship between ABSI and AAC, identifying a critical threshold (K-point) at 0.0908. However, further exploration through weighted smoothed curve fitting and threshold effect analysis revealed a significant nonlinear relationship between ABSI and ln-transformed AAC scores, identifying a critical threshold point at 0.0908 (**Figure 2**). Below this threshold, no significant association was observed between ABSI and AAC (for ln(AAC8 + 1): β=3.16, 95% CI: –1.40–7.71, P = 0.1748; for ln(AAC24 + 1): β=4.27, 95% CI: –2.53–11.07, P = 0.2184). Above the threshold, however, increasing ABSI was associated with a marked increase in AAC burden (for ln(AAC8 + 1): β=37.25, 95% CI: 10.16–64.34, P = 0.0071; for ln(AAC24 + 1): β=47.99, 95% CI: 7.58–88.40, P = 0.0200). Log-likelihood ratio tests confirmed the statistical significance of this threshold effect (P = 0.018 for ln(AAC8 + 1), P = 0.042 for ln(AAC24 + 1)) (**Table 3**).

**Table 2:** Associations between ABSI and ln(AAC8+1), ln(AAC24+1).

ABSI	Model1 *β*(95%CI) *P* value	Model2 *β*(95%CI) *P* value	Model3 *β*(95%CI) *P* value
Ln(AAC8+1)	24.74 (20.74, 28.74) <0.0001	8.43 (4.19, 12.68) 0.0001	5.05 (0.77, 9.33) 0.0208
T1	Ref	Ref	Ref
T2	0.10 (0.05, 0.14) <0.0001	0.03 (-0.01, 0.08) 0.1263	0.02 (-0.02, 0.06) 0.3660
T3	0.25 (0.20, 0.29) <0.0001	0.08 (0.03, 0.12) 0.0016	0.04 (-0.01, 0.08) 0.1138
*P* for trend	<0.0001	0.0016	0.1136
ln(AAC24+1)	38.65 (32.62, 44.68) <0.0001	12.10 (5.76, 18.45) 0.0002	6.70 (0.32, 13.08) 0.0397
T1	Ref	Ref	Ref
T2	0.16 (0.09, 0.23) <0.0001	0.05 (-0.01, 0.12) 0.1082	0.03 (-0.03, 0.09) 0.3434
T3	0.39 (0.33, 0.46) <0.0001	0.11 (0.04, 0.18) 0.0013	0.06 (-0.01, 0.13) 0.1194
*P* for trend	<0.0001	0.0013	0.1190

Model 1: variables were not adjusted. Model 2: adjustments were made to age, gender, and race. Model 3: Fully adjusted for age, gender, race, education level, poverty income ratio (PIR), hypertension, diabetes, smoking status, and alcohol intake.

T, tertiles; PIR, Ratio of family income to poverty; BMI, Body mass index; AAC, abdominal aortic calcification.

**Table 3:** Threshold effect analysis of ABSI on AAC.

Outcome	ln(AAC8+1)	ln(AAC24+1)
Model3		
*β* (95%CI) P value	5.0500 (0.7711, 9.3289) 0.0208	6.6983 (0.3171, 13.0794) 0.0397
Model3^+^		
threshold point	0.0908	0.0908
*β*1(<0.0908)	3.1554 (-1.4008, 7.7117) 0.1748	4.2689 (-2.5275, 11.0652) 0.2184
*β*2(> 0.0908)	37.2507 (10.1586, 64.3428) 0.0071	47.9891 (7.5772, 88.4009) 0.0200
* β*2-*β*1	34.0953 (5.7687, 62.4219) 0.0184	43.7202 (1.4669, 85.9735) 0.0426
AAC at threshold point	0.4918 (0.4522, 0.5313)	0.7439 (0.6842, 0.8036)
Logarithmic likelihood ratio test *P* value	0.018	0.042

Fully adjusted for age, gender, race, education level, poverty income ratio (PIR), hypertension, diabetes, smoking status, and alcohol intake.

PIR, Ratio of family income to poverty; HBP, High blood pressure; AAC, abdominal aortic calcification.

We conducted subgroup analyses by age, sex, race, smoking, alcohol consumption, HBP, and diabetes mellitus to explore the relationship between ABSI and AAC in different populations (**Table 4**). After adjusting for covariates, only age significantly modified the association between ABSI and ln-transformed AAC scores. Specifically, a much stronger positive correlation was evident among participants aged 60 years and older (for ln(AAC8 + 1): β=21.64, 95% CI: 15.37–27.91, P<0.0001; for ln(AAC24 + 1): β=31.14, 95% CI: 21.77–40.51, P<0.0001).

**Table 4:** Subgroup analysis of the associations between ABSI and AAC.

Subgroup	ln(AAC8+1) *β*(95%CI) *P* value	*P* for interaction	ln(AAC24+1) *β*(95%CI) *P* value	*P* for interaction
Age		<0.0001		<0.0001
<60	1.72 (-4.12, 7.56) 0.5642		3.57 (-5.16, 12.30) 0.4224	
≥60	21.64 (15.37, 27.91) <0.0001		31.14 (21.77, 40.51) <0.0001	
Gender		0.8800		0.8229
Male	5.29 (-1.49, 12.07) 0.1263		7.40 (-2.72, 17.52) 0.1518	
Female	4.63 (-0.82, 10.08) 0.0961		5.94 (-2.20, 14.07) 0.1526	
Race		0.6966		0.8294
Mexican American	-4.24 (-21.17, 12.69) 0.6238		-5.62 (-30.86, 19.62) 0.6625	
Other Hispanic	4.74 (-17.94, 27.42) 0.6819		6.53 (-27.28, 40.33) 0.7052	
Non-Hispanic White	5.80 (0.79, 10.81) 0.0233		7.67 (0.20, 15.14) 0.0442	
Non-Hispanic Black	-1.68 (-15.14, 11.79) 0.8072		-1.88 (-21.95, 18.20) 0.8545 0.9014	
Non-Hispanic Asian	13.93 (-6.12, 33.99) 0.1734		15.29 (-14.60, 45.19) 0.3161	
Other Race	7.69 (-27.73, 43.11) 0.6704		15.75 (-37.04, 68.55) 0.5587	
Alcohol intakes ≥12drinks/year		0.7149		0.7033
Yes	5.28 (0.38, 10.17) 0.0346		7.04 (-0.26, 14.33) 0.0587	
No	3.43 (-5.30, 12.16) 0.4417		4.16 (-8.85, 17.18) 0.5308	
Smoked ≥ 100 cigarettes		0.2088		0.2170
Yes	8.08 (1.54, 14.63) 0.0155		11.31 (1.56, 21.06) 0.0231	
No	2.55 (-3.13, 8.24) 0.3784		3.22 (-5.25, 11.69) 0.4566	
HBP		0.5778		0.5409
Yes	6.24 (0.08, 12.40) 0.0473		8.63 (-0.55, 17.82) 0.0655	
No	3.83 (-2.08, 9.74) 0.2044		4.68 (-4.12, 13.49) 0.2974	
Diabetes		0.1189		0.1406
Yes	12.16 (2.21, 22.11) 0.0167		16.71 (1.87, 31.55) 0.0273	
No	3.53 (-1.13, 8.20) 0.1380		4.56 (-2.40, 11.52) 0.1994	

Fully adjusted for age, gender, race, education level, poverty income ratio (PIR), hypertension, diabetes, smoking status, and alcohol intake.

T, tertiles; PIR, Ratio of family income to poverty; HBP, High blood pressure; AAC, abdominal aortic calcification.

## 4 Discussion

This research examined the correlation between ABSI and AAC among 3077 middle-aged and elderly individuals in the United States. The results of the study showed that both AAC24 and AAC8 maintained a significant positive correlation with ABSI, and the two were nonlinearly correlated with a K-point of 0.0908, with a non-significant correlation to the left of the K-point and a significant positive correlation to the right of the K-point. This positive correlation suggests that as the ABSI increases, the AAC also increases, especially after the K-point, which may suggest the potential value of the ABSI in assessing the risk of cardiovascular disease.

This investigation marks a pioneering effort to probe the relationship between ABSI and AAC. Traditional reliance on BMI for obesity assessment has been challenged by BMI’s limited capacity to distinguish between different types of body fat and its strong correlation with waist circumference, underscoring BMI’s inadequacies in obesity diagnosis. The recognition of central obesity as a crucial element in cardiovascular risk evaluation led to the creation of ABSI. Since its introduction in 2012, ABSI has been linked to a range of diseases and metabolic disorders, with elevated ABSI values signaling a heightened risk of premature mortality in the broader population (11). ABSI’s effectiveness in detecting visceral and muscle-reducing obesity in overweight or obese adults with Type 2 Diabetes Mellitus (T2DM) underscores its superiority over BMI in predicting diabetes and chronic kidney disease (CKD) in certain populations (14, 15). The direct relationship between ABSI and cardiovascular risk, as well as its stronger correlation with mortality (overall, cardiovascular, and cancer), highlights its potential as a predictor of cardiovascular events (16–18). Our results suggest that individuals with a higher ABSI may have more abdominal fat accumulation, which is associated with increased AAC scores. Abdominal fat is considered a metabolically active tissue that secretes a variety of inflammatory factors and hormones, which may be one of the mechanisms that promote atherosclerosis (19). Thus, ABSI could offer a straightforward and accessible measure for clinicians to identify middle-aged and older adults at elevated risk of developing AAC and subsequent cardiovascular diseases. Furthermore, the determination of K-points offers a clinical benchmark, suggesting potential thresholds for intensified monitoring and intervention efforts.

The positive correlation between ABSI and AAC may involve complex biological mechanisms. The systemic inflammatory state induced by obesity is a key factor in the promotion of atherosclerosis and vascular calcification (20, 21). Inflammatory mediators released from adipose tissue, such as tumor necrosis factor α (TNF-α) and interleukin 6 (IL-6), exacerbate inflammatory responses in the vascular wall, which further contribute to the calcification process (20, 22). At the same time, obesity is closely related to insulin resistance and T2DM (23), and these pathological states raise the risk of vascular calcification by increasing calcium salt deposition in vascular smooth muscle cells (24–26). In addition, obesity is strongly associated with abnormalities of lipid metabolism, such as hypertriacylglycerolemia and high low-density lipoprotein (LDL) cholesterol levels (27, 28). These abnormalities have been suggested to be the major contributing factors to both atherosclerosis and vascular calcification (29). Obesity also leads to an increase in oxidative stress (30), which may disrupt vascular endothelial function and promote inflammatory responses as well as the migration and proliferation of vascular smooth muscle cells, all of which are critical aspects of the calcification process (31). Hormonal imbalances induced by obesity, such as altered levels of lipofuscin and leptin, also have a direct or indirect effect on vascular calcification. Of these, leptin may promote calcification due to its proinflammatory properties (32–34). Whereas lipocalin, which has anti-inflammatory effects, is reduced in obese individuals and may increase the risk of calcification (35–37). Vitamin D deficiency is more common in obese individuals (38, 39), and vitamin D is essential for calcium and phosphorus metabolism and maintenance of vascular health. Its deficiency may promote vascular calcification through various mechanisms (40–42). In summary, inflammation, insulin resistance and diabetes mellitus, abnormal lipid metabolism, oxidative stress, adipose-related hormone imbalances, and vitamin D deficiency constitute a complex network of interactions that together drive the progression of vascular calcification.

Subgroup analyses and interaction tests revealed significant modifications in the relationship between the ABSI and AAC by age and diabetes status. Specifically, a more pronounced positive correlation between ABSI and AAC was evident among participants aged 60 years and older. This finding implies that central obesity’s impact on cardiovascular disease (CVD) risk escalates with advancing age. Such a trend could be attributed to a blend of age-associated biological changes, including genetic and potentially epigenetic factors, environmental influences like diabetes mellitus and chronic kidney disease, and the propensity of vascular smooth muscle cells to adopt an osteogenic phenotype — key contributors to age-dependent vascular calcification (43). Furthermore, processes inherent to the aging phenomenon, such as cellular senescence, autophagy, the emission of extracellular vesicles, and oxidative stress, play pivotal roles in facilitating vascular calcification (44). Additionally, within the diabetes subgroups, a notable correlation between ABSI and AAC was observed among patients with borderline blood glucose levels. However, this association might indicate statistical variance arising from the smaller sample sizes of the subgroups under examination.

This study utilized data collected by NHANES between 2013 and 2014 and included 3,077 adults aged 40 years and older as study participants. This large and diverse sample makes our findings more representative and generalizable. Methodologically, we employed sophisticated statistical analysis techniques, such as multivariate linear regression, smooth curve fitting, threshold effect analysis, subgroup analysis, and interaction test, to delve into the interactions between ABSI and AAC. This methodology was utilized to ensure the accuracy and reliability of the study results. This study pays special attention to the potential association between ABSI and AAC that has not been fully explored, providing a new perspective for understanding the link between obesity and cardiovascular disease risk.

However, this study also has some limitations. As a cross-sectional study, it failed to establish a causal relationship between ABSI and AAC, although it revealed a correlation between the two. Future studies may need to employ prospective or interventional designs to explore this relationship in depth. Additionally, the data sources relied upon for this study were limited to NHANES data from 2013 to 2014, which may not fully reflect current population health status and trends. Finally, although this study considered multiple covariates, it may have still missed other potential variables, such as lifestyle and dietary habits, which may also influence the relationship between ABSI and AAC.

## Data Availability

The original contributions presented in the study are included in the article/supplementary material. Further inquiries can be directed to the corresponding author.
