# Correction: Association between adjusted body size index and abdominal aortic calcification among US older adults aged 40 years and above from a cross-sectional survey of the NHANES 2013–2014

**DOI:** 10.3389/fendo.2026.1868170

**Published:** 2026-06-15

**Authors:** Yutong Chen, Yi Ding, Shanliang Jin, Yanwei Zhang

**Affiliations:** Department of Anesthesiology, Shanghai Ninth People’s Hospital, Shanghai Jiao Tong University School of Medicine, Shanghai, China

**Keywords:** adjusted body size index, abdominal aortic calcification, cross-sectional survey, elderly, NHANES

The title of this article was erroneously given as: “Association between a body shape index and cognitive impairment among US older adults aged 40 years and above from a cross-sectional survey of the NHANES 2011-2014”. The correct title of the article is “Association between adjusted body size index and abdominal aortic calcification among US older adults aged 40 years and above from a cross-sectional survey of the NHANES 2013-2014”.

There was a mistake in **Figure 1** as published. **Figure 1** of the original published paper is about the participants from 2011 to 2014. The corrected **Figure 1** and its caption appear below.

**Figure 1:**
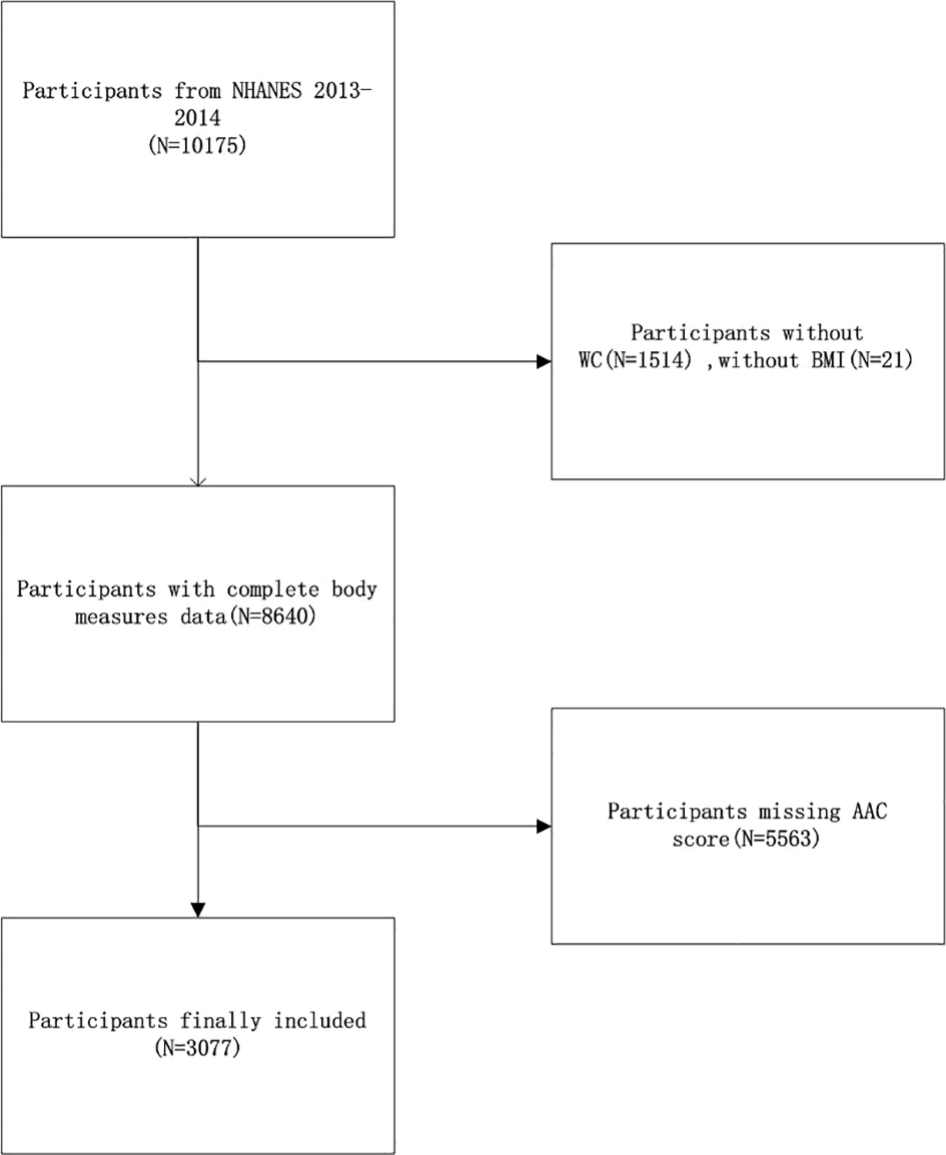
Flow chart showing the NHANES participants' selection.

There were mistakes in **Tables 2**, **3**, and **4** as published. The original tables reported regression coefficients based on untransformed AAC scores and contained incorrect statistical formatting. The corrected tables, which now report coefficients based on natural log-transformed AAC scores (ln(AAC8 + 1) and ln(AAC24 + 1)) and use standardized statistical notation appear below.

**Table 2:** Associations between ABSI and ln(AAC8+1), ln(AAC24+1).

ABSI	Model1 *β*(95%CI) *P* value	Model2 *β*(95%CI) *P* value	Model3 *β*(95%CI) *P* value
Ln(AAC8+1)	24.74 (20.74, 28.74) <0.0001	8.43 (4.19, 12.68) 0.0001	5.05 (0.77, 9.33) 0.0208
T1	Ref	Ref	Ref
T2	0.10 (0.05, 0.14) <0.0001	0.03 (-0.01, 0.08) 0.1263	0.02 (-0.02, 0.06) 0.3660
T3	0.25 (0.20, 0.29) <0.0001	0.08 (0.03, 0.12) 0.0016	0.04 (-0.01, 0.08) 0.1138
*P* for trend	<0.0001	0.0016	0.1136
ln(AAC24+1)	38.65 (32.62, 44.68) <0.0001	12.10 (5.76, 18.45) 0.0002	6.70 (0.32, 13.08) 0.0397
T1	Ref	Ref	Ref
T2	0.16 (0.09, 0.23) <0.0001	0.05 (-0.01, 0.12) 0.1082	0.03 (-0.03, 0.09) 0.3434
T3	0.39 (0.33, 0.46) <0.0001	0.11 (0.04, 0.18) 0.0013	0.06 (-0.01, 0.13) 0.1194
*P* for trend	<0.0001	0.0013	0.1190

Model 1: variables were not adjusted. Model 2: adjustments were made to age, gender, and race. Model 3: Fully adjusted for age, gender, race, education level, poverty income ratio (PIR), hypertension, diabetes, smoking status, and alcohol intake.

T, tertiles; PIR, Ratio of family income to poverty; BMI, Body mass index; AAC, abdominal aortic calcification.

**Table 3:** Threshold effect analysis of ABSI on AAC.

Outcome	ln(AAC8+1)	ln(AAC24+1)
Model3		
*β* (95%CI) P value	5.0500 (0.7711, 9.3289) 0.0208	6.6983 (0.3171, 13.0794) 0.0397
Model3^+^		
threshold point	0.0908	0.0908
*β*1(<0.0908)	3.1554 (-1.4008, 7.7117) 0.1748	4.2689 (-2.5275, 11.0652) 0.2184
*β*2(> 0.0908)	37.2507 (10.1586, 64.3428) 0.0071	47.9891 (7.5772, 88.4009) 0.0200
*β*2-*β*1	34.0953 (5.7687, 62.4219) 0.0184	43.7202 (1.4669, 85.9735) 0.0426
AAC at threshold point	0.4918 (0.4522, 0.5313)	0.7439 (0.6842, 0.8036)
Logarithmic likelihood ratio test *P* value	0.018	0.042

Fully adjusted for age, gender, race, education level, poverty income ratio (PIR), hypertension, diabetes, smoking status, and alcohol intake.

PIR, Ratio of family income to poverty; HBP, High blood pressure; AAC, abdominal aortic calcification.

**Table 4:** Subgroup analysis of the associations between ABSI and AAC.

Subgroup	ln(AAC8+1) *β*(95%CI) *P* value	*P* for interaction	ln(AAC24+1) *β*(95%CI) *P* value	*P* for interaction
Age		<0.0001		<0.0001
<60	1.72 (-4.12, 7.56) 0.5642		3.57 (-5.16, 12.30) 0.4224	
≥60	21.64 (15.37, 27.91) <0.0001		31.14 (21.77, 40.51) <0.0001	
Gender		0.8800		0.8229
Male	5.29 (-1.49, 12.07) 0.1263		7.40 (-2.72, 17.52) 0.1518	
Female	4.63 (-0.82, 10.08) 0.0961		5.94 (-2.20, 14.07) 0.1526	
Race		0.6966		0.8294
Mexican American	-4.24 (-21.17, 12.69) 0.6238		-5.62 (-30.86, 19.62) 0.6625	
Other Hispanic	4.74 (-17.94, 27.42) 0.6819		6.53 (-27.28, 40.33) 0.7052	
Non-Hispanic White	5.80 (0.79, 10.81) 0.0233		7.67 (0.20, 15.14) 0.0442	
Non-Hispanic Black	-1.68 (-15.14, 11.79) 0.8072		-1.88 (-21.95, 18.20) 0.8545 0.9014	
Non-Hispanic Asian	13.93 (-6.12, 33.99) 0.1734		15.29 (-14.60, 45.19) 0.3161	
Other Race	7.69 (-27.73, 43.11) 0.6704		15.75 (-37.04, 68.55) 0.5587	
Alcohol intakes ≥12drinks/year		0.7149		0.7033
Yes	5.28 (0.38, 10.17) 0.0346		7.04 (-0.26, 14.33) 0.0587	
No	3.43 (-5.30, 12.16) 0.4417		4.16 (-8.85, 17.18) 0.5308	
Smoked ≥ 100 cigarettes		0.2088		0.2170
Yes	8.08 (1.54, 14.63) 0.0155		11.31 (1.56, 21.06) 0.0231	
No	2.55 (-3.13, 8.24) 0.3784		3.22 (-5.25, 11.69) 0.4566	
HBP		0.5778		0.5409
Yes	6.24 (0.08, 12.40) 0.0473		8.63 (-0.55, 17.82) 0.0655	
No	3.83 (-2.08, 9.74) 0.2044		4.68 (-4.12, 13.49) 0.2974	
Diabetes		0.1189		0.1406
Yes	12.16 (2.21, 22.11) 0.0167		16.71 (1.87, 31.55) 0.0273	
No	3.53 (-1.13, 8.20) 0.1380		4.56 (-2.40, 11.52) 0.1994	

Fully adjusted for age, gender, race, education level, poverty income ratio (PIR), hypertension, diabetes, smoking status, and alcohol intake.

T, tertiles; PIR, Ratio of family income to poverty; HBP, High blood pressure; AAC, abdominal aortic calcification.

Throughout the published article, there were inconsistencies in the formatting of statistical values and symbols. All statistical symbols (P, β) have now been properly italicized, and all confidence intervals now use en dashes (–) instead of hyphens (-) in accordance with standard scientific publishing guidelines.

In the published article, there was an error in the statistical analysis description. The original manuscript failed to disclose that natural logarithmic transformation was applied to the non-normally distributed AAC scores to meet the assumptions of linear regression, and incorrectly reported regression coefficients based on untransformed AAC values.

A correction has been made to Section 2.5 *Statistical analysis*, Paragraph 2. This paragraph previously stated:

“Participants were divided into three groups of equal size based on their ABSI scores, and disparities in demographic attributes were examined using chi-square tests for categorical variables and t-tests for continuous variables. To assess the link between ABSI and AAC, a multivariate linear regression model was employed.”

The corrected paragraph appears below:

“Participants were divided into three equal tertiles by ABSI scores. Demographic differences were examined using chi-square tests for categorical variables and weighted t-tests for continuous variables. Since AAC scores were non-normally distributed with many zero values, we applied natural logarithmic transformation (ln(AAC8 + 1) and ln(AAC24 + 1)) to the outcome variables, which satisfied the normality assumption of linear regression. Multivariate linear regression was used to assess the association between ABSI and AAC. All regression coefficients reported in the results are based on the natural log-transformed AAC scores.”

In the published article, there was an ambiguity regarding covariate adjustment. The original manuscript did not explicitly state that education level was included as a covariate in all fully adjusted multivariable models.

A correction has been made to Section 2.4 *Covariates*, adding the following sentence at the end of the paragraph:

“All these covariates were included in the fully adjusted multivariable regression models.”

3. Missing Data Handling

In the published article, there was an omission in the description of missing data handling. The original manuscript did not report the method used to address missing covariate values.

A correction has been made to Section 2.4 *Covariates*, adding the following sentences:

“Missing data were present in several covariates with an overall missing rate of 0.76%. We performed random forest imputation using the R package missForest to handle missing values, which has been shown to outperform traditional methods such as mean imputation and multiple imputation for small amounts of missing data. Sensitivity analysis using complete-case analysis yielded nearly identical results (data not shown). After imputation, the dataset was complete, and all analyses were based on the fully imputed data.”

In the published article, there was an error in the reported regression coefficients and their interpretation. The original manuscript incorrectly reported coefficients based on untransformed AAC scores and used the clinically meaningless “per 1 unit increase in ABSI” metric.

A correction has been made to Section 3.2 *Relationship between ABSI and AAC*, Paragraph 1. This paragraph previously stated:

“In Model 3, which accounted for all covariates, each unit increase in ABSI corresponded to a 26.62-point increase in the AAC24 score [β=26.62, (0.42, 52.82), p=0.0465]. Moreover, a positive correlation between ABSI and AAC8 scores was consistently observed across all models. Specifically, in Model 1, the correlation coefficient was β=56.46 (47.33, 65.58); in Model 2, it was β=19.51 (9.83, 29.19); and in Model 3, it was β=11.69 (1.93, 21.46) with a p-value of 0.0190, indicating statistical significance.”

The corrected paragraph appears below:

“In Model 3, which additionally included education level, poverty income ratio (PIR), hypertension, diabetes, smoking status, and alcohol intake, each 0.01 unit increase in ABSI was associated with a 0.0505 increase in ln(AAC8 + 1) (β=5.05 per 0.01 unit, 95% CI: 0.77–9.33, P = 0.0208) and a 0.0670 increase in ln(AAC24 + 1) (β=6.70 per 0.01 unit, 95% CI: 0.32–13.08, P = 0.0397). When ABSI was divided into tertiles, no significant linear trend was observed in the fully adjusted model.”

In the published article, there was an error in the terminology used for the threshold effect analysis and the reported coefficients. The original manuscript used the non-standard term “K-point” and reported coefficients based on untransformed AAC scores.

A correction has been made to Section 3.2 *Relationship between ABSI and AAC*, Paragraph 2. This paragraph previously stated:

“However, further exploration through smoothed curve fitting and threshold effect analysis revealed a nonlinear relationship between ABSI and AAC, identifying a critical threshold (K-point) at 0.0908 (Figure 2). The left and right side effects of AAC24 at the K-points were 5.42 (-23.08, 33.93) 0.7093, and 289.22 (146.25, 432.18) <0.0001, respectively; AAC8 had 4.59 (-6.04, 15.22) 0.3976 and 99.68 (46.35, 153.01) 0.0003 left- and right-side effects at the K-points, respectively, with log-likelihood-ratio tests of less than 0.001(Table 3).”

The corrected paragraph appears below:

“However, further exploration through weighted smoothed curve fitting and threshold effect analysis revealed a significant nonlinear relationship between ABSI and ln-transformed AAC scores, identifying a critical threshold point at 0.0908 (Figure 2). Below this threshold, no significant association was observed between ABSI and AAC (for ln(AAC8 + 1): β=3.16, 95% CI: –1.40–7.71, P = 0.1748; for ln(AAC24 + 1): β=4.27, 95% CI: –2.53–11.07, P = 0.2184). Above the threshold, however, increasing ABSI was associated with a marked increase in AAC burden (for ln(AAC8 + 1): β=37.25, 95% CI: 10.16–64.34, P = 0.0071; for ln(AAC24 + 1): β=47.99, 95% CI: 7.58–88.40, P = 0.0200). Log-likelihood ratio tests confirmed the statistical significance of this threshold effect (P = 0.018 for ln(AAC8 + 1), P = 0.042 for ln(AAC24 + 1)) (Table 3).”

In the published article, there was an error in the subgroup analysis results. The original manuscript incorrectly reported that diabetes status significantly modified the association between ABSI and AAC.

A correction has been made to Section 3.2 *Relationship between ABSI and AAC*, Paragraph 3. This paragraph previously stated:

“We conducted subgroup analyses by subgroups of age, sex, race, smoking, alcohol consumption, HBP, and diabetes mellitus to explore the relationship between ABSI and AAC in different populations (Table 4). After adjusting for covariates, significant differences in the relationship between ABSI and AAC were found in subgroups with different ages and diabetes status.”

The corrected paragraph appears below:

“We conducted subgroup analyses by age, sex, race, smoking, alcohol consumption, HBP, and diabetes mellitus to explore the relationship between ABSI and AAC in different populations (Table 4). After adjusting for covariates, only age significantly modified the association between ABSI and ln-transformed AAC scores. Specifically, a much stronger positive correlation was evident among participants aged 60 years and older (for ln(AAC8 + 1): β=21.64, 95% CI: 15.37–27.91, P<0.0001; for ln(AAC24 + 1): β=31.14, 95% CI: 21.77–40.51, P<0.0001).”

The original version of this article has been updated.

